# Immunohistochemical analysis indicates that the anatomical location of B-cell non-Hodgkin’s lymphoma is determined by differentially expressed chemokine receptors, sphingosine-1-phosphate receptors and integrins

**DOI:** 10.1186/s40164-015-0004-3

**Published:** 2015-04-01

**Authors:** Stephen Middle, Sarah E Coupland, Azzam Taktak, Victoria Kidgell, Joseph R Slupsky, Andrew R Pettitt, Kathleen J Till

**Affiliations:** Department of Molecular and Clinical Cancer Medicine, University of Liverpool, Liverpool, England; Medical Physics and Clinical Engineering, Royal Liverpool University Hospital, Liverpool, England; ORLAU, RJAH Orthopaedic hospital NHS Foundation Trust, Oswestry, England

**Keywords:** B-NHL, Lymphoma, Integrin, Chemokine, Homing, S1P receptors, Egress, Microenvironment

## Abstract

**Background:**

The aim of this study was to elucidate the mechanisms responsible for the location of B-cell non-Hodgkin’s lymphoma (B-NHL) at different anatomical sites. We speculated that the malignant B cells in these disorders have the potential for trafficking between blood and secondary lymphoid organs (SLO) or extranodal sites and that their preferential accumulation at different locations is governed by the expression of key molecules that regulate the trafficking of normal lymphocytes.

**Methods:**

Biopsy or blood samples from 91 cases of B-NHL affecting SLO (n = 27), ocular adnexae (n = 51) or blood (n = 13) were analysed by immunohistochemistry or flow cytometry for the expression of the following molecules: CCR7, CCL21 and αL (required for the entry of normal lymphocytes into SLO); CXCR4, CXCL12 and α4 (required for entry into extranodal sites); CXCR5, CXCL13 and S1PR2 (required for tissue retention); S1PR1 and S1PR3 (required for egress into the blood). The expression of each of these molecules was then related to anatomical location and histological subtype.

**Results:**

The expression of motility/adhesion molecules varied widely between individual patient samples and correlated much more strongly with anatomical location than with histological subtype. SLO lymphomas [comprising 10 follicular lymphoma (FL), 8 diffuse large B-cell lymphoma (DLBCL), 4 mantle-cell lymphoma (MCL) and 5 marginal-zone lymphoma (MZL)] were characterised by pronounced over-expression of S1PR2, suggesting that the malignant cells in these lymphomas are actively retained at the site of clonal expansion. In contrast, the malignant B cells in ocular adnexal lymphomas (10 FL, 9 DLBCL, 4 MCL and 28 MZL) expressed a profile of molecules suggesting a dynamic process of trafficking involving not only tissue retention but also egress via S1PR3 and homing back to extranodal sites via CXCR4/CXCL12 and α4. Finally, leukaemic lymphomas (6 FL, 5 MCL and 2 MZL) were characterised by aberrant expression of the egress receptor S1PR1 and low expression of molecules required for tissue entry/retention.

**Conclusions:**

In summary, our study strongly suggests that anatomical location in B-NHL is governed by the differential expression of specific adhesion/motility molecules. This novel observation has important implications for therapeutic strategies that aim to disrupt protective micro-environmental interactions.

**Electronic supplementary material:**

The online version of this article (doi:10.1186/s40164-015-0004-3) contains supplementary material, which is available to authorized users.

## Background

Lymphocytes are motile cells owing to their pivotal role in immune surveillance. They traffic through the immune system in search of the specific antigen that binds to their unique antigen receptor. However, the lymphocytes of B-cell non-Hodgkin’s lymphomas (B-NHL) tend to arise and become lodged within tissues [1] and in this way differ from their normal counterparts. The mechanisms underlying the homing and accumulation of lymphoma cells in secondary lymphoid organs (SLO) (i.e. lymph nodes (LN) and the spleen) and extranodal sites are not known. Since the tissue microenvironment provides growth and survival stimuli for both normal [2-4] and neoplastic lymphocytes [5-7], understanding the mechanisms involved in the tissue residency of the neoplastic lymphocytes in B-NHL could lead to new therapeutic approaches.

**Figure 1:**
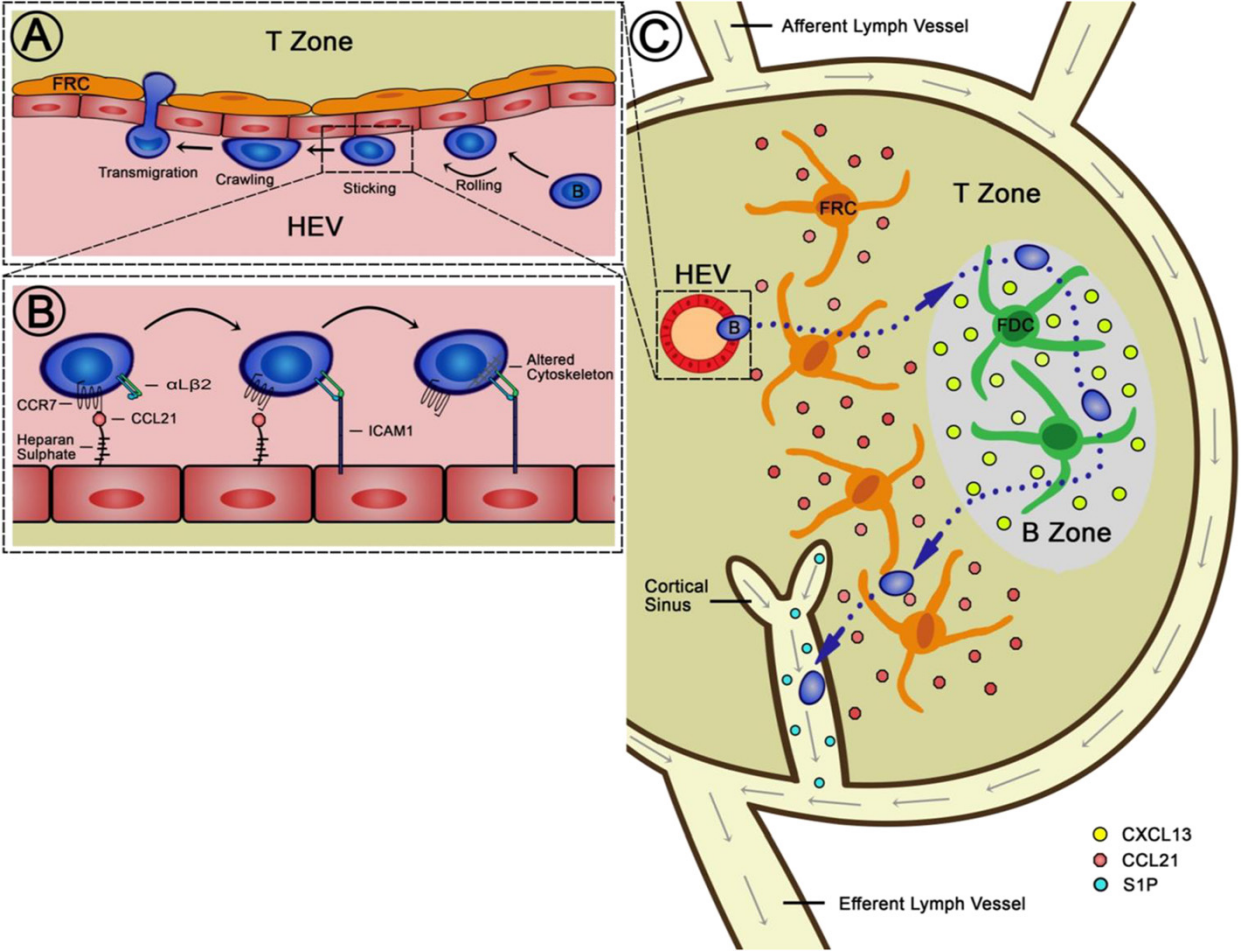
The role of adhesion molecules, chemokines and S1P in lymphocyte entry, egress and retention. (**A**) B-cell migration into lymph nodes via high endothelial venules (HEVs) is achieved through a multistep adhesion process involving rolling, sticking, crawling and finally transmigration. (**B**). Movement towards the chemokine CCL21 into nodes is dependent on the integrin αLβ2 binding to ICAM-1 on the HEV. (**C**) Once in the node B cells move in the direction of the highest concentration of chemokine/S1P. Movement towards the B zone of the nodes is dependent on the chemokine CXCL13 and/or α4β1. The direction of movement is shown here as a linear track, however cells within the lymph node go on a ‘random walk’ visiting the B and T cell zones multiple times in the search for antigen before exiting through the lymphatic sinuses in a S1PR1 dependent manner independently of integrin engagement.

We hypothesised that the trafficking and tissue localisation of lymphoma cells are governed by the expression of molecules responsible for the homing of normal lymphocytes, i.e. chemokines, chemokine and sphingosine-1-phosphate (S1P) receptors, and integrins. The chemokines CXCL12 (SDF-1), CXCL13 (BLC), and CCL21 (SLC) promote entry of B cells into tissues by binding to their respective receptors CXCR4, CXCR5 (BCA-1) and CCR7 [3,8,9]. In addition to its role in tissue entry, CXCL13 directs B cells into the primary and secondary follicles of LN (Figure 1) and the spleen [10,11]. Chemokines also activate adhesion molecules, which facilitate the trafficking of lymphocytes into and within tissues. The lymphocyte integrins, αLβ2 (LFA-1) and α4β1 (VLA-4), bind to their respective ligands on vascular endothelium and are important for transendothelial migration into LN (Figure 1) and extranodal tissues, respectively [10,12,13]. In addition, α4β1 mediates adhesion to fibronectin, which is involved in the trafficking and adhesion of lymphocytes within tissues [14,15].

Regarding the exit (egress) of lymphocytes from the LN and spleen, this process is controlled by S1PR1 (Figure 1) and S1PR3, respectively [16,17]. In contrast, S1PR2 has an inhibitory role within lymphoid organs, preventing B-cells from responding to chemokine signalling within the follicular region [18], resulting in their retention in the germinal centre (GC) [19]. The importance of S1PR2 in lymphoma is underlined by the fact that the gene is mutated in approximately 25% of cases of diffuse large B cell lymphoma (DLBCL) [20]. Furthermore, about 50% of mice with targeted disruption of S1PR2 develop lymphoma [21].

Although the expression of some of these molecules has been examined in some types of lymphoma [13,22-24], present knowledge is incomplete and difficult to comprehend, as chemokine/S1P receptors and integrins act together in a co-ordinated way to determine lymphocyte localisation and trafficking. We therefore conducted a comprehensive and systematic analysis of relevant chemokine receptors, S1PRs and integrins in B-NHLs affecting different anatomical sites, exploiting the fact that, although most B-NHL occur within SLO [25], up to 25% occur at extranodal sites [26,27], such as the ocular adnexa [12], and in the blood [1]. By applying a combination of immunohistochemical and flow cytometric approaches to B-NHL located in SLO, ocular adnexae and blood (leukaemic lymphomas), we found that B-NHL in the three different tissue compartments expressed distinct patterns of lymphocyte homing molecules, which could explain their tissue localisation.

## Results and discussion

### Relationship between anatomical location and lymphoma subtype

In order to relate the expression of molecules involved in lymphocyte adhesion and migration to the anatomical location and lymphoma subtype, it was first important to understand the relationship between the latter two variables within the cohort of patients selected for this study. As is shown in Additional file 1: Table S1, among the 27 lymphomas cases of the SLO, twice as many had a diagnosis of FL (10) or DLBCL (8) than of MZL (5) or MCL (4). In contrast, amongst the 51 OAL, more than half were EMZL (28), with FL (10) and DLBCL (9) accounting for most of the remaining cases, while the majority of the 13 leukaemic lymphomas were either FL (6) or MCL (5). Looking at the relationship between the lymphoma subtype and the anatomical location the other way round, among cases of FL or MCL, the proportion with SLO, ocular adnexal or blood involvement was similar (10 versus 10 versus 6 and 4 versus 4 versus 5, respectively). In contrast, whilst DLBCL occurred with similar frequency in the SLO or ocular adnexa (8 versus 9, respectively), it rarely involved the blood. For MZL, 80% of the 35 cases presented as OAL. The positive association between MZL and OAL and negative association between DLBCL and leukaemic lymphoma reached statistical significance (P < 0.001), whereas a weaker association was evident between MCL and leukaemic presentation in this cohort.

### Expression of molecules involved in entry of lymphocytes into LN

**Figure 2:**
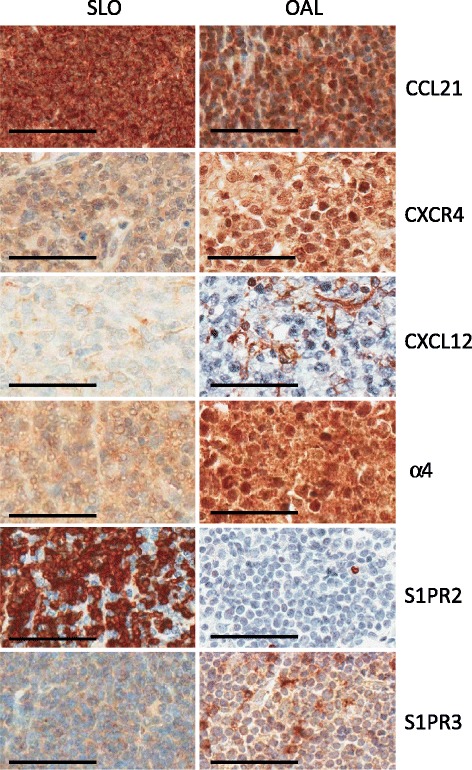
Representative cases of SLO and OAL showing different expression of molecules involved in lymphocyte homing. TMA cores for CCL21, S1PR2 and S1PR3 from MCL; CXCR4, CXCL12 and α4 are from DLBCL. (Other histological types are shown in Additional file 2: Figure S1). Bar = 60 μM.

**Figure 3:**
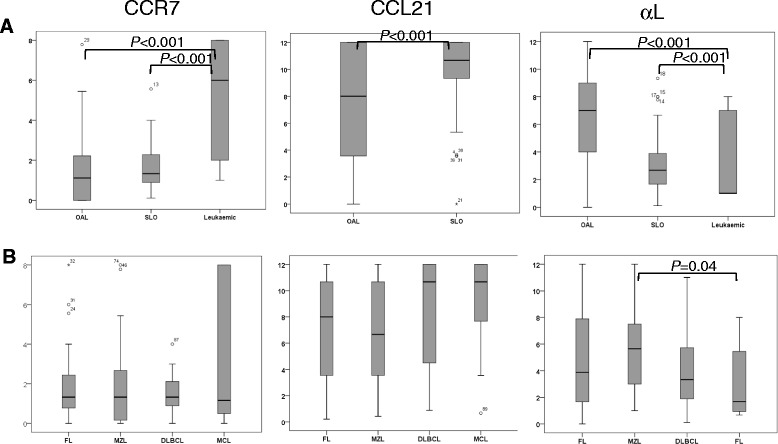
Expression of molecules involved in entry into SLO. **A**. Box and whiskers plots show lymphomas analysed according by tissue of origin. **B**. Box and whiskers plots show lymphomas according to histology. Statistically significant differences in expression are shown.

In order to elucidate the mechanisms responsible for the preferential localisation of lymphomas at different anatomical sites, we first analysed the molecules involved in the entry of lymphocytes into LN, namely CCR7, its ligand CCL21 and the integrin αLβ2. The cases were initially grouped according to anatomical location irrespective of lymphoma subtype (Figures 2 and 3A; Table 1A). CCR7 was expressed at low levels in lymphomas affecting the SLO, ocular adnexae and blood, the highest expression being observed in leukaemic lymphomas (P < 0.001). In contrast, CCL21 was expressed at much higher levels in lymphomas affecting the SLO compared to OAL (P < 0.001), thereby providing greater potential for receptor-ligand interaction and, consequently, tissue entry. αL was expressed by lymphomas affecting all three anatomical sites but at different levels, being highest in OAL (P < 0.001) and lowest in leukaemic lymphomas (P < 0.001). Comparison between individual lymphoma entities irrespective of anatomical location (Figure 3B; Table 1B) showed that the expression of CCR7, CCL21 and αL was remarkably constant, although levels of αL were higher in MZL than MCL, in keeping their respective associations with OAL and leukaemic lymphoma.

**Table 1:** **Expression of molecules involved in lymphocyte trafficking**

**A. Anatomical sites**											
**Lymphoma subtype**	**Entry into SLO**			**Entry into extranodal tissues**			**Retention in tissues**			**Egress**	
	**CCR7**	**CCL21**	**αL**	**CXCR4**	**CXCL12**	**α4**	**CXCR5**	**CXCL13**	**S1PR2**	**S1PR1**	**S1PR3**
SLO	±	+++	+	++	±	+	++	±	+++	±	+
OAL	±	+	+++	+++	+	+++	+++	+	-	±	++
LL	+		±	±		±	±		-	+++	NA
**B. Histological features**											
**Lymphoma subtype**	**Entry into SLO**			**Entry into extranodal tissues**			**Retention in tissues**			**Egress**	
	**CCR7**	**CCL21**	**αL**	**CXCR4**	**CXCL12**	**α4**	**CXCR5**	**CXCL13**	**S1PR2**	**S1PR1**	S1PR3*
FL	±	+++	+	+	+	++	++	+	±	±	+
MZL	±	+++	++	+++	+	++	++	+	-	+	+
DLBCL	±	+++	+	++	+	++	+++	+	++	-	+
MCL	±	+++	±	++	-	++	++	+	±	++	+

Expression is based on the median staining scores: 0–1 (−); >1-2 (±); >2-4 (+); >4-6 (++); >6 (+++). SLO - secondary lymphoid organ; OAL – ocular adnexal lymphoma; LL - leukaemic lymphoma; NA - not available.

Statistically significant differences in the expression of CCR7, CCL21 and αL were not observed when lymphomas were compared by anatomical site within each histological subtype, or when they were compared by subtype within each anatomical site (Additional file 2: Figure S1 and 2A). However, the small number of cases in some of the groups precludes firm conclusions from being drawn. Analysis of different anatomical variants of OAL showed no association between CCR7, CCL21 or αL expression and location in the eyelid, conjunctiva or orbit (Additional file 2: Figure S2B).

### Expression of molecules involved in entry into extranodal sites

**Figure 4:**
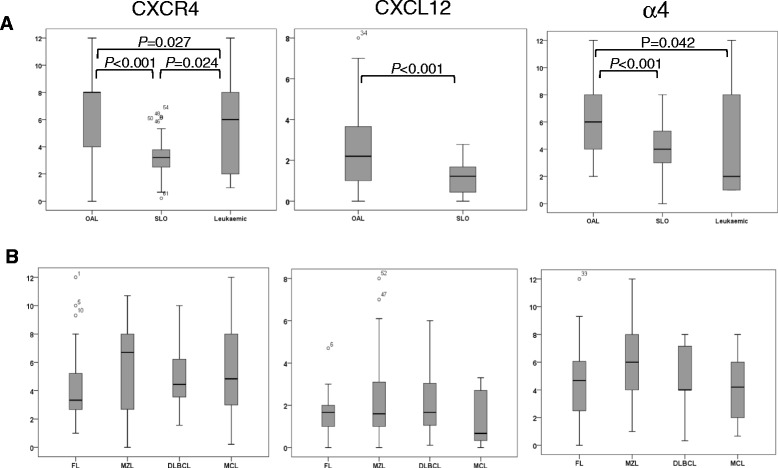
Expression of molecules involved in entry into extranodal sites. **A**. Box and whiskers plots show lymphomas analysed according by tissue of origin. **B**. Box and whiskers plots show lymphomas according to histology. Statistically significant differences in expression are shown.

We next analysed the molecules involved in the entry of lymphocytes into extranodal tissues, namely CXCR4, CXCL12 and α4 integrin. Grouping cases according to anatomical location (Figures 2 and 4A, Table 1A) showed that CXCR4 and α4 were expressed at significantly higher levels in OAL compared to lymphomas affecting the SLO or blood, indicating a greater potential for migration into extranodal sites. Furthermore, CXCL12 was present at higher levels in OAL compared to lymphomas of the SLO, thereby providing a stronger homing signal for CXCR4-expressing cells. Leukaemic lymphomas expressed particularly low levels of both CXCR4 and α4. Comparison between individual lymphoma subtype irrespective of anatomical location (Figure 4B and Additional file 2: Figure S3A; Table 1B) revealed no significant differences in the expression of any of these molecules, although there was a trend towards higher CXCR4 expression in MZL and lower CXCL12 expression in MCL, in keeping with their respective associations with OAL and leukaemic lymphoma.

Statistically significant differences in the expression of CXCR4, CXCL12 and α4 were not observed when lymphomas were compared by anatomical site within each histological subtype, or when they were compared by subtype within each anatomical site (Additional file 2: Figure S3A). However, the small number of cases in some of the groups precludes firm conclusions from being drawn. Analysis of anatomical variants of OAL showed that CXCL12 was expressed at higher levels in cases located in the eyelid as opposed to the conjunctiva or orbit (p < 0.014) (Additional file 2: Figure S3B).

### Expression of molecules involved in retention within tissues

**Figure 5:**
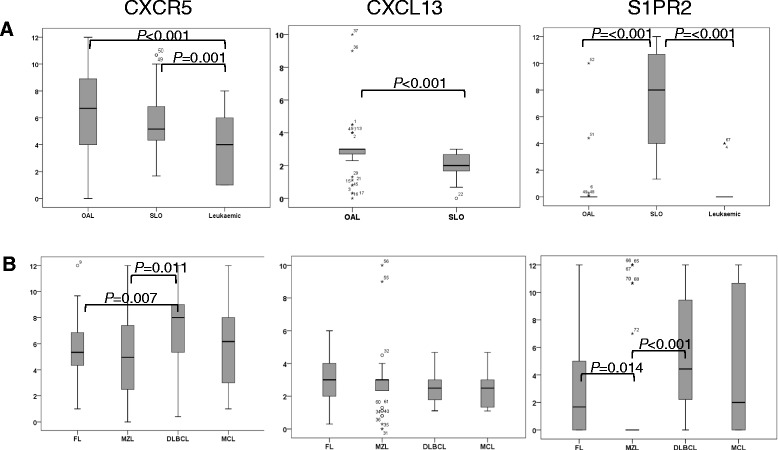
Expression of molecules involved in retention within LN. **A**. Box and whiskers plots show lymphomas analysed according by tissue of origin and **B**. Box and whiskers plots show lymphomas according to histology. Statistically significant differences in expression are shown.

Having analysed the molecules involved in the entry of lymphocytes into tissues, we next turned our attention to factors involved primarily in retention within tissues, i.e. CXCR5, CXCL13 and S1PR2. Grouping lymphomas according to anatomical location (Figure 2 and 5A, Table 1A) showed that CXCR5 was expressed at higher levels in lymphomas affecting the SLO and ocular adnexae (P < 0.001) than in leukaemic lymphomas (P < 0.001). CXCL13 was also expressed, albeit at low levels, in ocular adnexal and SLO lymphomas, expression being higher in OAL (P < 0.001). Of particular note, S1PR2 was expressed at very high levels in lymphomas of the SLO but was virtually absent in OAL (P < 0.001) and leukaemic lymphomas (P < 0.001). This profile of CXCR5, CXCL13 and S1PR2 expression strongly suggests that the malignant B-cells in lymphomas of the SLO (and to a lesser extent OAL) are actively retained in the tissues, whereas the malignant cells in leukaemic lymphoma are allowed unimpeded passage through the tissues and back into the blood. Comparison between individual histological subtypes irrespective of anatomical location (Figure 5B; Table 1B) revealed no significant differences in the expression of CXCL13. However, levels of CXCR5 were highest in DLBCL in keeping with its negative association with leukaemic lymphoma, whereas S1PR2 expression was lowest in MZL in keeping with the fact that only a minority of these cases affected the SLO.

Statistically significant differences in the expression of CXCR5, CXCL13 and S1PR2 were not observed when lymphomas were compared by anatomical site within each histological subtype (Additional file 2: Figure S4A). However, the small number of cases in some of the groups precludes firm conclusions from being drawn. Comparing cases by histological subtype within each anatomical site showed differences in CXCR5 and S1PR2 expression among lymphomas of the SLO only. Specifically, CXCR5 expression was highest in nodal DLBCL and lowest in MZL (Additional file 2: Figure S4A). These findings were in keeping with those in the overall cohort.

Analysis of anatomical variants of OAL indicated that there were no significant differences in the expression of CXCR5, CXCL13 and S1PR2 between lymphomas located in the orbit, conjunctiva or lid (Additional file 2: Figure S4B).

### Expression of molecules involved in egress from tissues

**Figure 6:**
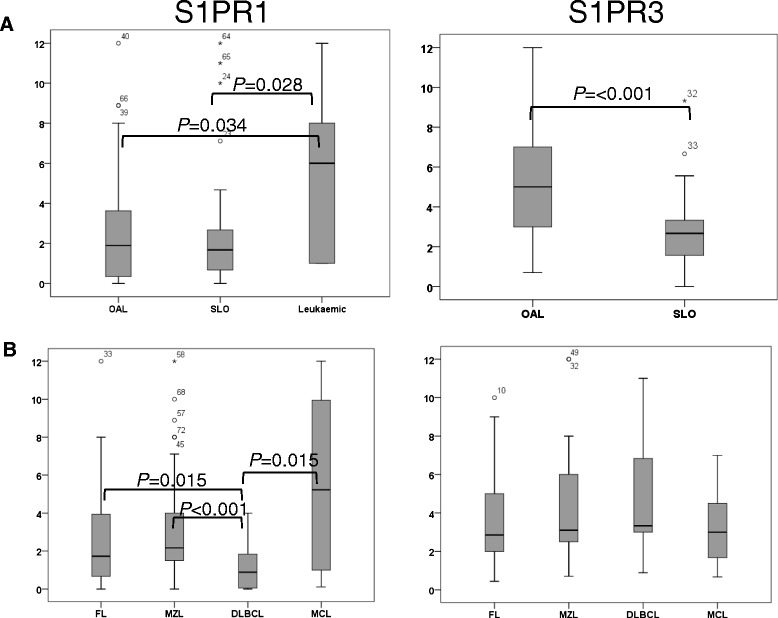
Expression of molecules involved in egress from tissues.** A**. Box and whiskers plots show lymphomas analysed according to histology and **A**. Lymphomas analysed according by tissue of origin. **B**. Box and whiskers plots show lymphomas according to histology. Statistically significant differences in expression are shown.

Finally, we analysed the egress receptors S1PR1 and S1PR3. Grouping lymphomas according to anatomical location (Figures 2 and 6A, Table 1A) revealed that S1PR1 was expressed at much higher levels in leukaemic lymphomas compared to OAL and lymphomas of the SLO. This result was unexpected given that S1PR1 is expressed at low levels in normal lymphocytes as a result of rapid internalisation of the receptor following binding to its ligand, which is present at high levels in the blood [28]. Technical problems with the antibody precluded flow cytometric analysis of S1PR3 in leukaemic lymphomas. However, S1PR3 was expressed at higher levels in OAL compared to lymphomas of the SLO. Comparison between histological subtype irrespective of anatomical location (Figure 6B; Table 1B) revealed mostly similar expression of S1PR1 and S1PR3 among the different lymphoma subtypes, although S1PR1 was expressed at low levels in DLBCL in keeping with its negative association with leukaemic lymphoma.

Statistically significant differences in the expression of S1PR1 and S1PR3 were not observed when lymphomas were compared by anatomical site within each histological subtype, or by lymphoma subtype within each anatomical site (Additional file 2: Figure S5A). However, the small number of cases in some of the groups precludes firm conclusions from being drawn.

Analysis of anatomical variants of OAL indicated that there were no significant differences in the expression of S1PR1 or S1PR3 between lymphomas located in the orbit, conjunctiva or lid (Additional file 2: Figure S5B).

### Overall correlation between anatomical location and expression of molecules involved in lymphocyte trafficking

The expression of most of the motility and adhesion molecules examined in this study varied widely between cases and correlated much more strongly with anatomical location than with lymphoma histology. Furthermore, where expression did correlate with lymphoma histology, this reflected an association between the specific histological entity and a particular anatomical location. For example, the expression profile of MZL could be explained by the association between MZL and OAL, whereas that of DLBCL could be explained by the negative association between DLBCL and leukaemic lymphoma. These observations indicate that the adhesion/motility molecules expressed in B-NHL are a feature of anatomical location rather than lymphoma histology.

### Potential explanation for the anatomical location of SLO lymphomas

Our study also provides compelling evidence that the motility and adhesion molecules expressed by different B-NHL are responsible for their different anatomical locations. In the case of lymphomas located in the SLO, our findings indicate that the neoplastic B cells may have difficulty in exiting from the tissues because they express relatively low levels of the egress receptors S1PR1 and S1PR3. Furthermore, the high expression of CXCR5 and S1PR2 by these cells should result in their active retention at the site of clonal expansion. Our demonstration that high CXCR5 expression is a feature of all types of lymphomas of the SLO, including nodal DLBCL, is in keeping with, and adds to, previous reports documenting high CXCR5 expression in FL, MCL and MZL [24,29,30], all of which frequently involve either the LN or the spleen. However, the relatively low tissue expression of CXCL13 in lymphomas of the SLO calls into question the functional significance of high CXCR5 expression and suggests that S1PR2 over-expression may play a dominant role in mediating tissue retention given the ubiquitous presence of its ligand in the blood and lymph. In addition to S1PR2-mediated tissue retention, the high levels of CCL21 expressed in lymphomas of the SLO should provide a tissue homing signal for any lymphoma cells that escape into the blood. However, the relatively low expression of αL in lymphomas of the SLO calls into question their ability to enter LNs effectively [31]. Taken together, therefore, the profile of motility and adhesion molecules expressed in lymphomas of the SLO suggests that the malignant cells are actively retained at the site of clonal expansion primarily due to high expression of S1PR2. This striking and unique feature of nodal and splenic lymphomas was not expected as mutations in the gene encoding S1PR2 have been reported in a significant proportion of patients with nodal DLBCL, and generally result in reduced expression of the receptor [20]. Further investigation of this apparent anomaly is required but was beyond the scope of the present study.

### Potential explanation for the anatomical location of OAL

The expression profile of motility and adhesion molecules in OAL was quite different from that in lymphomas of the SLO, and included high expression of α4 and CXCR4 together with significant levels of CXCL12. This combination of features should favour the entry of neoplastic B-cells cells into extranodal sites since α4β1 is essential for migration across the endothelium of small veins towards the sites of inflammation [32], while CXCR4 has been implicated in the dissemination of many malignancies to multiple different tissues [33,34]. 7Interestingly, OAL cells also expressed high levels of αL, which is involved in entry into LN. However, OALs expressed CCR7 at very low levels, calling into question their ability to migrate into tissues expressing CCL21. In common with lymphomas of the SLO, OALs expressed high levels of CXCR5. They also expressed detectable levels of CXCL13, suggesting the potential for ligand-receptor interactions favouring tissue retention. With regard to S1P receptors, OAL cells did not express significant levels of S1PR2 or S1PR1 but did express high levels of S1PR3 suggesting that these cells have the potential for egress into the blood. Taken together, these findings suggest that the malignant cells in OAL likely undergo a dynamic process of trafficking involving egress into the blood (directed by S1PR3) and homing back to the extranodal tissues (directed by CXCR4/CXCL12 and mediated by α4) where an element of retention may occur (via CXCR5/CXCL13). This concept is entirely in keeping with the fact that OAL are frequently bilateral [35] and that extranodal lymphomas often progress to involve multiple extranodal tissues, including not only the conjunctiva but also lacrimal glands, salivary glands, stomach, lung, thyroid gland and skin [26,27]. The initial confinement of many cases of extranodal lymphoma to a single anatomical site is possibly explained by their evolution on a background of site-specific chronic inflammation. For example, OAL can be associated with underlying autoimmune disease (e.g. Sjogren’s syndrome) [35] and, in some geographical areas, Chamydia psittaci infections [35,36]. However, once established, extranodal lymphomas are likely to acquire the potential to disseminate to multiple sites owing to their trafficking properties.

### Potential explanation for the anatomical location of leukaemic lymphomas

In addition to explaining the anatomical location of tissue-based lymphomas, our study also helps to explain why some cases of B-NHL present with leukaemia. Thus, the neoplastic B cells from patients with leukaemic lymphoma expressed low or insignificant levels of molecules required for entry into SLO (CCR7, αL), entry into extranodal tissues (CXCR4, α4) and tissue retention (CXCR5, S1PR2). In contrast, they expressed very high levels of the egress receptor S1PR1. This expression profile strongly favours egress over tissue entry/retention and provides a plausible explanation for the accumulation of the malignant cells in the blood. It is noteworthy that the expression all of these molecules in leukaemic lymphomas differs from their expression in normal blood B cells [37-40]. Thus S1PR1 is not found on circulating normal B cells due to the abundance of its ligand, S1P, in plasma resulting in receptor internalisation [28]. Although we did not measure S1P in the blood, there are no reports in the literature of levels of this ubiquitous glycolipid being reduced in any disease state. Therefore, the high expression of S1PR1 on the neoplastic B cells of leukaemic lymphomas can be regarded as aberrant. The same consideration applies to the low expression of CXCR5, since normal circulating B cells express high levels of this receptor [41]. Elucidating the mechanisms responsible for the aberrant over-expression of S1PR1 and under-expression of CXCR5 in leukaemic lymphomas was beyond the scope of the present study but is the subject of ongoing investigation. The low expression of α4 on leukaemic lymphomas is also at odds with its ubiquitous expression on normal B cells but is in keeping with its frequent absence in chronic lymphocytic leukaemia [31].

## Conclusion

The development and progression of B-NHL is known to depend on interactions with the tumour microenvironment that are mediated/directed by chemokines, chemokine receptors and integrins [42]. Consequently, by elucidating the molecules and likely processes responsible for the location of lymphomas at different anatomical sites, our study has implications for both existing and emerging therapies. The protective effect of the microenvironment in the context of existing treatments is illustrated by a recent report on follicular lymphoma, which traced the clones responsible for relapse following chemotherapy. These clones had the same genetic fingerprint as the original clone and had been able to survive in the tissues during clinical remission [43]. With regard to emerging therapies, drugs such as ibrutinib and idelalisib, which inhibit kinases involved in B-cell receptor signalling, have successfully been used in the treatment of lymphoma [44-46]. One of the striking effects of these drugs is displacement of malignant B cells from their protective environmental niches, and available evidence suggests that this effect results at least in part from blockade of chemokine-induced adhesion signals [47]. Our study suggests that a similar effect might be achieved through the targeted blockade of specific chemokine receptors. In keeping with this concept, inhibitors of CXCR4 [48] or CXCR5 [49] have recently been shown to enhance the efficacy of rituximab therapy in a mouse model of B-NHL.

In summary, our study constitutes the first systematic attempt to explain why malignant lymphocytes in B-NHL accumulate at different anatomical sites. We showed that most of the key molecules involved in the trafficking of normal lymphocytes are variably expressed and that their pattern of expression can explain the differential anatomical location of lymphomas in SLO, ocular adnexae and blood. In making these observations, our study indicates possible new approaches to therapy based on disrupting interactions between lymphoma cells and their protective micro-environment.

## Materials and methods

### Patients

All samples used for this study were obtained with informed consent. Formalin fixed, paraffin embedded tissue samples were obtained from 27 patients with lymphomas of SLO [10 follicular lymphoma (FL), 8 DLBCL, 4 mantle-cell lymphoma (MCL), 3 splenic marginal-zone lymphoma (SMZL) and 2 nodal MZL] and 51 patients with ocular adnexa lymphomas (OAL). The 51 OAL comprised 28 extranodal marginal zone B-cell lymphomas (EMZL), 10 FL, 9 DLBCL, and 4 MCL. Cryopreserved mononuclear cells were obtained from 13 patients with B-NHL who presented with blood involvement (6 FL, 5 MCL and 2 MZL). Clinical data linked to the lymphoma samples are shown in Additional file 1: Table S2.

### Tissue microarray (TMA) construction

Tissue cores of 0.6 mm diameter and were assembled into microarrays using the Manual Tissue Arrayer MTA-1 (Beecher instruments, WI, USA). Two to three cores from each donor tissue block were used. Colon, kidney and tonsil cores were incorporated into the TMA as control tissues.

### Antibodies

The following monoclonal antibodies were used: CXCR4, CXCL13, CCL21, S1PR3 and αL (Abcam, Cambridge, UK); CXCR5, CXCL12 (R&D Systems, Oxford, UK); CCR7 (Novus Biologicals, Cambridge, UK); S1PR1, α4 (Santa Cruz, Middlesex, UK); S1PR2 (Sigma, Poole, UK). Mouse IgG1 and rabbit Ig were used as negative controls (R&D Systems, Abingdon, UK). All antibodies were titrated on normal tissues and used at saturating concentrations (Additional file 1: Table S3).

### Antigen detection and measurement

#### Immunohistochemical staining

The EnVision™ staining method was used. As previously described, de-waxing of the sections and antigen retrieval were performed with EnVision™ FLEX target retrieval solution with the Dako PT-link module [50]. Slides were stained with an autostainer using the EnVision™ FLEX convenience kit (Dako, Cambridgeshire). Slides were counterstained in Meyers’ haematoxylin (Sigma).

#### TMA scoring

A standardised scoring method was used based on the percentage of lymphocytes showing any staining combined with the staining intensity [50]. A minimum of two cores from each tissue block were independently scored by at least two researchers (SM, KJT, SEC). The scores for the percentage of positive lymphocytes were as follows: none (0), 1-24% (1), 25-49% (2), 50-74% (3) and 75-100% (4). The intensity of the stained lymphocytes was scored as weak (1), moderate (2) or strong (3). Multiplying the percentage of stained lymphocytes by the staining intensity gave a protein expression score ranging from 0 (negative) to 12 (highest).

#### Flow cytometry

Blood lymphocytes were stained using an indirect immunofluorescence technique. Lymphoma B cells were identified by co-staining with CD19-PerCP-Cy5.5 (Becton Dickinson). The percentage and mean fluorescence intensity (MFI) were determined on CD19^+^ cells. The results were converted into a score as for the TMAs [for MFI [0–5 (0), 5–10 (1), 10–90 (2), >90 (3)].

### Statistical analysis

The distribution of chemokines and their receptors was first examined for normality using histograms and normal Q-Q plots. Where there was obvious deviation from normality, logarithm or square root transformations were applied.

For data that could be approximated using a normal distribution, family-wise comparison was carried out using one-way ANOVA. Where statistical significance was suggested at the 0.05 level, post-hoc pair-wise comparisons were carried out using the Tukey test.

## Additional files

Additional file 1:
**Supplementary Tables S1-S3.**
Additional file 2:
**Supplementary Figure S1-S5.**
