# Supplementary material for: Immunohistochemical analysis indicates that the anatomical location of B-cell non-Hodgkin’s lymphoma is determined by differentially expressed chemokine receptors, sphingosine-1-phosphate receptors and integrins
Source: Exp Hematol Oncol. 2015 Apr 1;4:10. doi: 10.1186/s40164-015-0004-3 (PMC4416323; doi:10.1186/s40164-015-0004-3)
Supplement: Additional file 1: — Supplementary Tables S1-S3. [file 40164_2015_4_MOESM1_ESM.docx]

**A. OAL**

|  | **Location** | | **Stage** |  |  | |  | |  |  |
| --- | --- | --- | --- | --- | --- | --- | --- | --- | --- | --- |
| **Disease** | **Within ocular adnexa** | **Side** | **Ann Arbour - diagnosis** | **TNM - diagnosis** | | **Age** | | **Sex** | **Treatment** | |
| DLBCL | Orbit with lacrimal | Right | IE | T2b | | 85 | | Female | Radiotherapy | |
| DLBCL | Lid | Bilateral | IE | T4c | | 66 | | Female | Radiotherapy and chemotherapy | |
| DLBCL | Orbit without lacrimal | Left | II | T2aN2 | | 74 | | Male | Radiotherapy and chemotherapy | |
| DLBCL | Orbit with lacrimal gland | Left | II | T2bN3 | | 64 | | Male | Radiotherapy and chemotherapy | |
| DLBCL | Orbit without lacrimal | Right | IE | T2a | | 73 | | Female | Radiotherapy | |
| DLBCL | Orbit without lacrimal | Right | IE | T2a | | 81 | | Male | Radiotherapy | |
| DLBCL | Orbit with lacrimal | Bilateral | IE | bT2b | | 77 | | Female | Radiotherapy and chemotherapy | |
| DLBCL | Orbit without lacrimal | Left | IE | T2b | | 63 | | Female | Radiotherapy and chemotherapy | |
| DLBCL | Lid | Right | IE | T3 | | 67 | | Male | Radiotherapy and chemotherapy | |
| EMZL | Lid | Right | IE | T3 | | 67 | | Male | Radiotherapy | |
| EMZL | Orbit without lacrimal | Right | IE | T2a | | 70 | | Female | Radiotherapy | |
| EMZL | Orbit with lacrimal gland | Left | IE | T2b | | 53 | | Male | Radiotherapy | |
| EMZL | Orbit with lacrimal gland | Left | IE | T2b | | 74 | | Female | Radiotherapy | |
| EMZL | Orbit without lacrimal | Left | IE | T2a | | 77 | | Female | Radiotherapy | |
| EMZL | Lid | Left | IE | T3 | | 79 | | Male | Radiotherapy and chemotherapy | |
| EMZL | Orbit without lacrimal | Bilateral | IE | T2a | | 91 | | Female | Radiotherapy and chemotherapy | |
| EMZL | Lid | Bilateral | IE | bT3 | | 91 | | Female | Radiotherapy and chemotherapy | |
| EMZL | Orbit without lacrimal | Right | IE | T2a | | 58 | | Female | Radiotherapy | |
| EMZL | Conjunctiva | Right | IE | T1a | | 79 | | Female | Excision only | |
| EMZL | Orbit without lacrimal | Right | IE | T2a | | 47 | | Female | Radiotherapy and chemotherapy | |
| EMZL | Lid | Left | IE | T3 | | 58 | | Female | Radiotherapy | |
| EMZL | Lid | Bilateral | IE | bT3 | | 76 | | Female | Radiotherapy and chemotherapy | |
| EMZL | Orbit without lacrimal | Bilateral | IE | bT2a | | 59 | | Female | Radiotherapy | |
| EMZL | Orbit without lacrimal | Right | IE | T2a | | 35 | | Male | Radiotherapy | |
| EMZL | Orbit with lacrimal | Bilateral | IE | bT2b | | 47 | | Male | Radiotherapy | |
| EMZL | Conjunctiva | Right | IE | T1b | | 60 | | Female | Excision only | |
| EMZL | Lid | Bilateral | IE | bT3 | | 65 | | Male | Radiotherapy | |
| EMZL | Orbit without lacrimal | Left | IIIE | T2aN3 | | 80 | | Male | Radiotherapy and chemotherapy | |
| EMZL | Conjunctiva | Left | IE | T1b | | 73 | | Male | Radiotherapy | |
| EMZL | Conjunctiva | Bilateral | IE | bT1b | | 83 | | Female | Excision and radiotherapy | |
| EMZL | Orbit without lacrimal | Right | IE | T2a | | 45 | | Female | Radiotherapy | |
| EMZL | Lid | Left | IE | T3 | | 45 | | Male | Radiotherapy | |
| EMZL | Conjunctiva | Right | IE | T1a | | 85 | | Female | Radiotherapy | |
| EMZL | Lid | Bilateral | IE | T3 | | 67 | | Male | Radiotherapy and chemotherapy | |
| EMZL | Orbit with lacrimal | Left | II | T2bN1 | | 62 | | Female | Radiotherapy and chemotherapy | |
| FL | Orbit with lacrimal | Right | IE | T2c | | 46 | | Female | Radiotherapy and chemotherapy | |
| FL | Conjunctiva | Left | IE | T1a | | 72 | | Female | Radiotherapy and chemotherapy | |
| FL | Orbit with lacrimal | Right | IE | T2b | | 70 | | Female | Radiotherapy and chemotherapy | |
| FL | Conjunctiva | Right | IE | T1b | | 82 | | Female | Excision and radiotherapy | |
| FL | Conjunctiva | Left | IE | T1c | | 42 | | Female | Radiotherapy | |
| FL | Orbit without lacrimal | Bilateral | IE | bT2b | | 56 | | Female | Radiotherapy and chemotherapy | |
| FL | Orbit without lacrimal | Right | II | T2aN1 | | 58 | | Male | Radiotherapy and chemotherapy | |
| FL | Orbit with lacrimal | Right | IE | T2bN0 | | 54 | | Female | Radiotherapy | |
| FL | Orbit without lacrimal | Right | III | T2aN2 | | 81 | | Male | Radiotherapy and chemotherapy | |
| FL | Orbit without lacrimal | Left | IIE | T2b | | 68 | | Female | Radiotherapy and chemotherapy | |
| MCL | Orbit without lacrimal | Left | IE | T2a | | 64 | | Male | Radiotherapy and chemotherapy | |
| MCL | Orbit without lacrimal | Left | IE | T2a | | 63 | | Male | Radiotherapy and chemotherapy | |
| MCL | Conjunctiva | Right | III | T1bN3 | | 65 | | Male | Radiotherapy and chemotherapy | |
| MCL | Lid | Right | II | T3N1 | | 67 | | Female | Radiotherapy and chemotherapy | |

**B. SLO**

| **Disease** | **Stage** | **Age** | **Sex** | **Treatment** |
| --- | --- | --- | --- | --- |
| DLBCL | III | 25 | Male | None |
| DLBCL | IIE | 68 | Male | None |
| DLBCL | IIE | 60 | Male | None |
| DLBCL | IIE | 76 | Male | None |
| DLBCL | IV | 45 | Male | None |
| DLBCL | IV | 74 | Female | None |
| DLBCL | III | 71 | Male | None |
| DLBCL | I | 76 | Female | Clb, pred, flud |
| DLBCL | IV | 73 | Male | Yes |
| FL | I | 59 | Female | None |
| FL | I | 66 | Female | None |
| FL | II/IIIA | 83 | Male | None |
| FL | III | 62 | Male | None |
| FL | II | 48 | Female | None |
| FL | II | 49 | Female | Radiotherapy and chemotherapy |
| FL | IV | 63 | Female | None |
| FL | I | 66 | Female | None |
| FL | III | 66 | Female | N.A |
| FL | I | 49 | Male | N.A |
| FL | I | 36 | Male | None |
| MCL | III | 70 | Male | R-CHOP |
| MCL | IV | 61 | Female | R-CHOP |
| MCL | IV | 78 | Female | N.A |
| MCL | III | 79 | Male | None |
| EMZL | IV | 63 | Male | None |
| MZL | IV | 73 | Male | None |
| SMZL | IV | 61 | Female | N.A |
| SMZL | I/II | 74 | Male | None |
| SMZL | I |  | Male | None |

**C. Leukemic Lymphomas**

| **Disease** | **Stage** | **Follow up** | **WBC** | **Age** | **Sex** | **Prior Treatment** |
| --- | --- | --- | --- | --- | --- | --- |
| FL | IV | 0 | 160.4 | 29 | Male | R-CHOP, Rituximab |
| FL | II | III | 79.8 | 52 | Female | None |
| FL | I | IB | 9.7 | 59 | Male | None |
| FL | III | Deceased | 12.3 | 73 | Male | None |
| FL | N.A | N.A | N.A | 79 | N.A | N.A |
| FL | II | N.A | N.A | 67 | Female | N.A |
| MCL | IV | N.A | 72.3 | 45 | Male | None |
| MCL | I | IV | 61 | 76 | Female | None |
| MCL | IV | Deceased | 25 | 79 | Male | FC, R-CHOP |
| MCL | N.A | N.A | 130 | 74 | Male | N.A |
| MCL | IV | Deceased | 37.6 | 68 | Male | R-CHOP |
| MZL | N.A | Deceased | 43 | 76 | Female | Splenectomy |
| MZL | IV | N.A | N.A | 71 | Male | Retuximab; Clb |

Supplementary Table 1**.** Patient details. Clb – chlorambucil; pred – methyl prednisolone; flu – fludarabine; R-CHOP – rituximab, cyclophosphamide, doxorubicin, vincristine, prednisolone. N.A. data not available.

| Antibody | Concentration (μg/ml) |
| --- | --- |
| CCR7 | 10 |
| CXCR4 | 10 |
| CXCR5 | 1 |
| CCL21 | 5 |
| CXCL12 | 25 |
| CXCL13 | 5 |
| S1PR1 | 4 |
| S1PR2 | 1 |
| S1PR3 | 23 |
| α4 | 4 |
| αL | 8.5 |

Supplementary Table 2. Concentrations of Antibodies used to stain TMAs.

|  | FL | DLBCL | MCL | MZL | Total |
| --- | --- | --- | --- | --- | --- |
| SLO lymphoma | 10 | 8 | 4 | 5 | 27 |
| OAL | 10 | 9 | 4 | 28 | 51 |
| Leukaemic lymphoma | 6 | 0 | 5 | 2 | 13 |
| Total | 26 | 17 | 13 | 35 | 91 |

Supplementary Table 3. Relationship between anatomical location and histological diagnosis.
