# Supplementary material for: Immunohistochemical analysis indicates that the anatomical location of B-cell non-Hodgkin’s lymphoma is determined by differentially expressed chemokine receptors, sphingosine-1-phosphate receptors and integrins
Source: Exp Hematol Oncol. 2015 Apr 1;4:10. doi: 10.1186/s40164-015-0004-3 (PMC4416323; doi:10.1186/s40164-015-0004-3)
Supplement: Additional file 2: — Supplementary Figure S1-S5. [file 40164_2015_4_MOESM2_ESM.pdf]

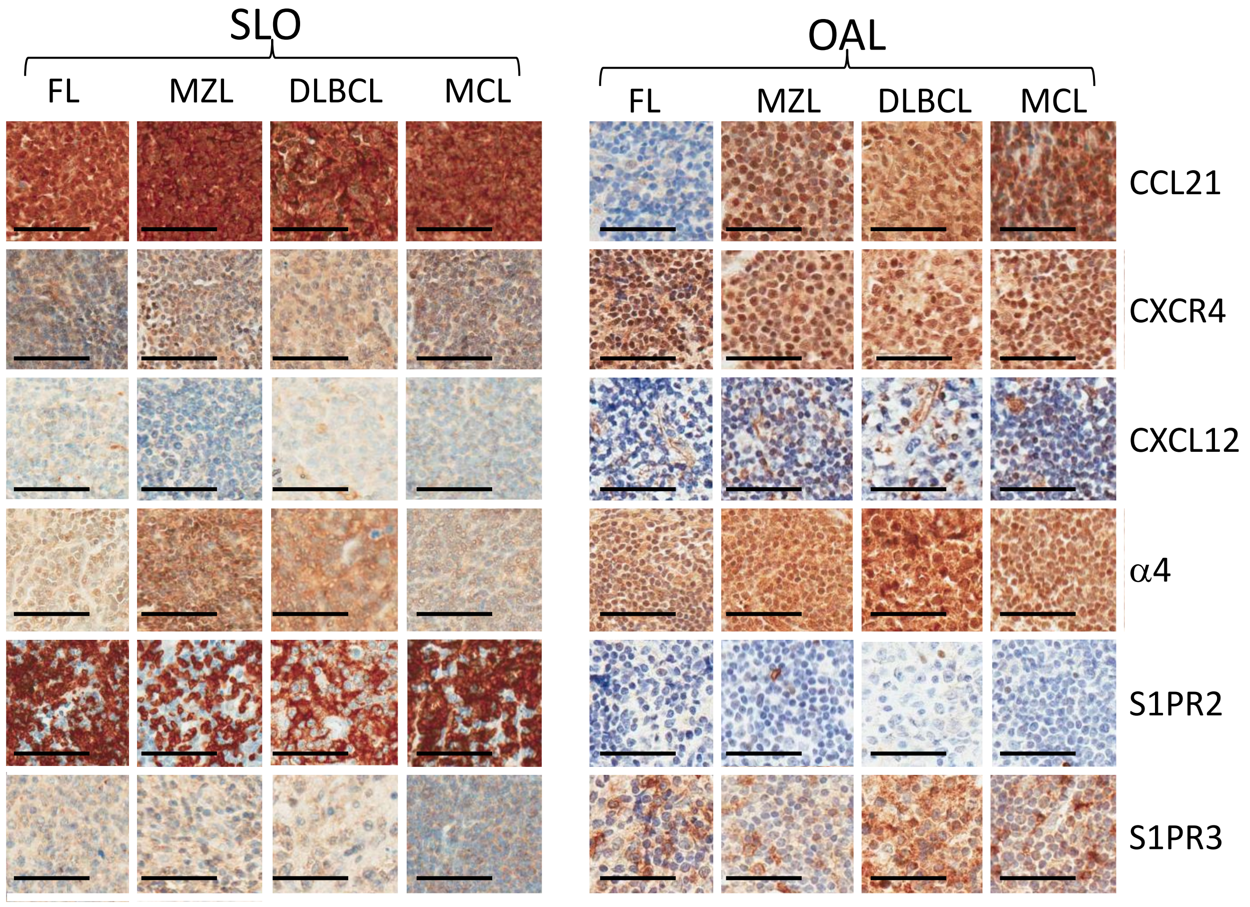

<u>Supplementary Figure 1. IHC of molecules involved in lymphoma cell homing.</u> Individual cores from lymphoma subtypes showing significantly different expression between ocular adnexa, and secondary lymphoid, organ lymphomas. Scale bars 60μm.

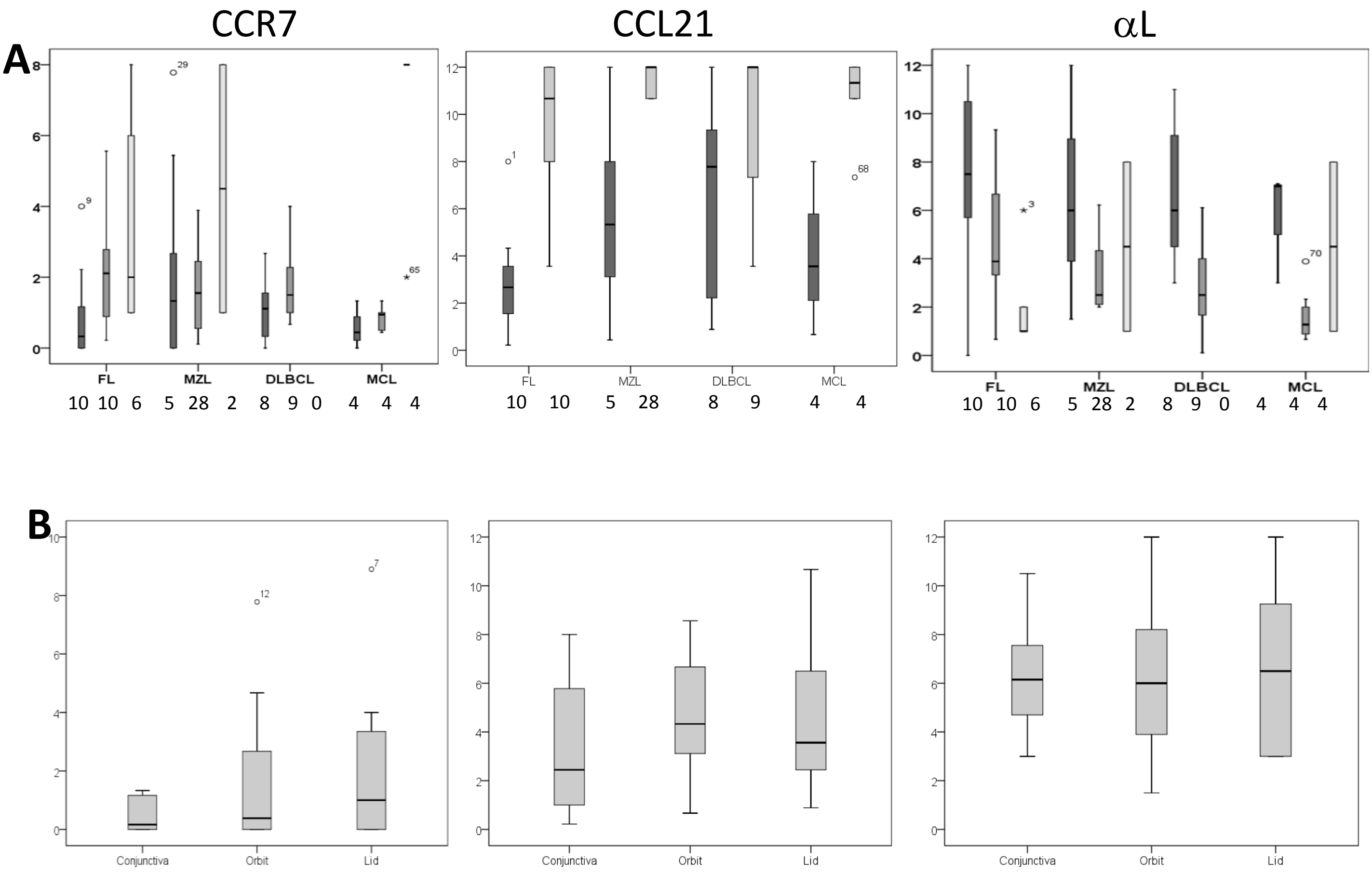

<u>Supplementary Figure 2. Expression of molecules involved in LN entry.</u> A. Box and whiskers plots of the individual histological subtypes in the ocular adnexa ■, SLO ■, and leukaemic □. Numbers below subtype indicate the number of patient samples. B. Box and whiskers plots of OAL at different locations within the ocular adnexa. No statistically significant differences were seen.

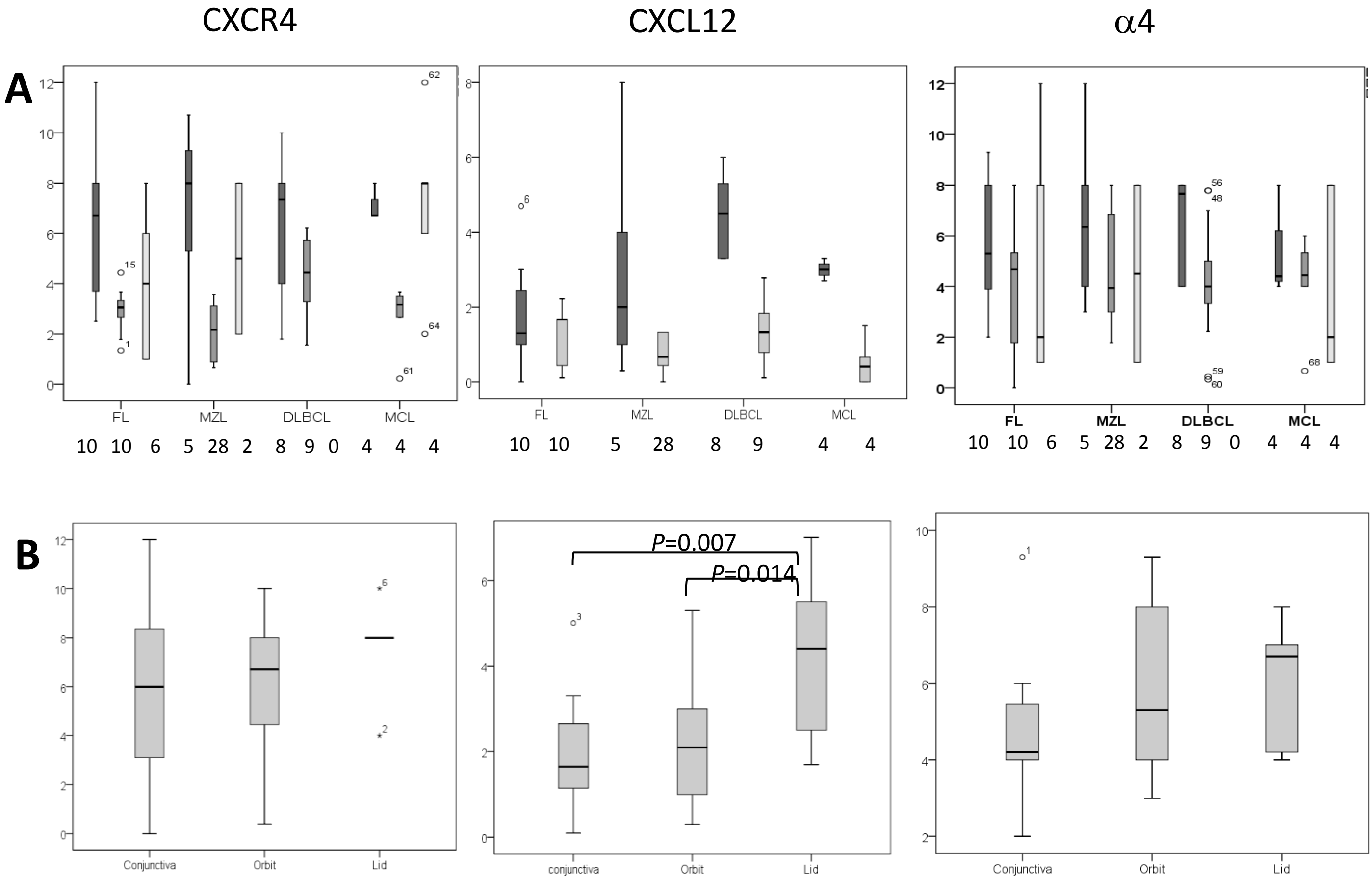

Supplementary Figure 3. Expression of molecules involved in entry into extranodal sites. A. Box and whiskers plots of the individual histological subtypes in the ocular adnexa ■, SLO ■, and leukaemic □. Numbers below subtype indicate the number of patient samples. B. Box and whiskers plots of OAL at different locations within the ocular adnexa. Statistically significant differences are annotated.

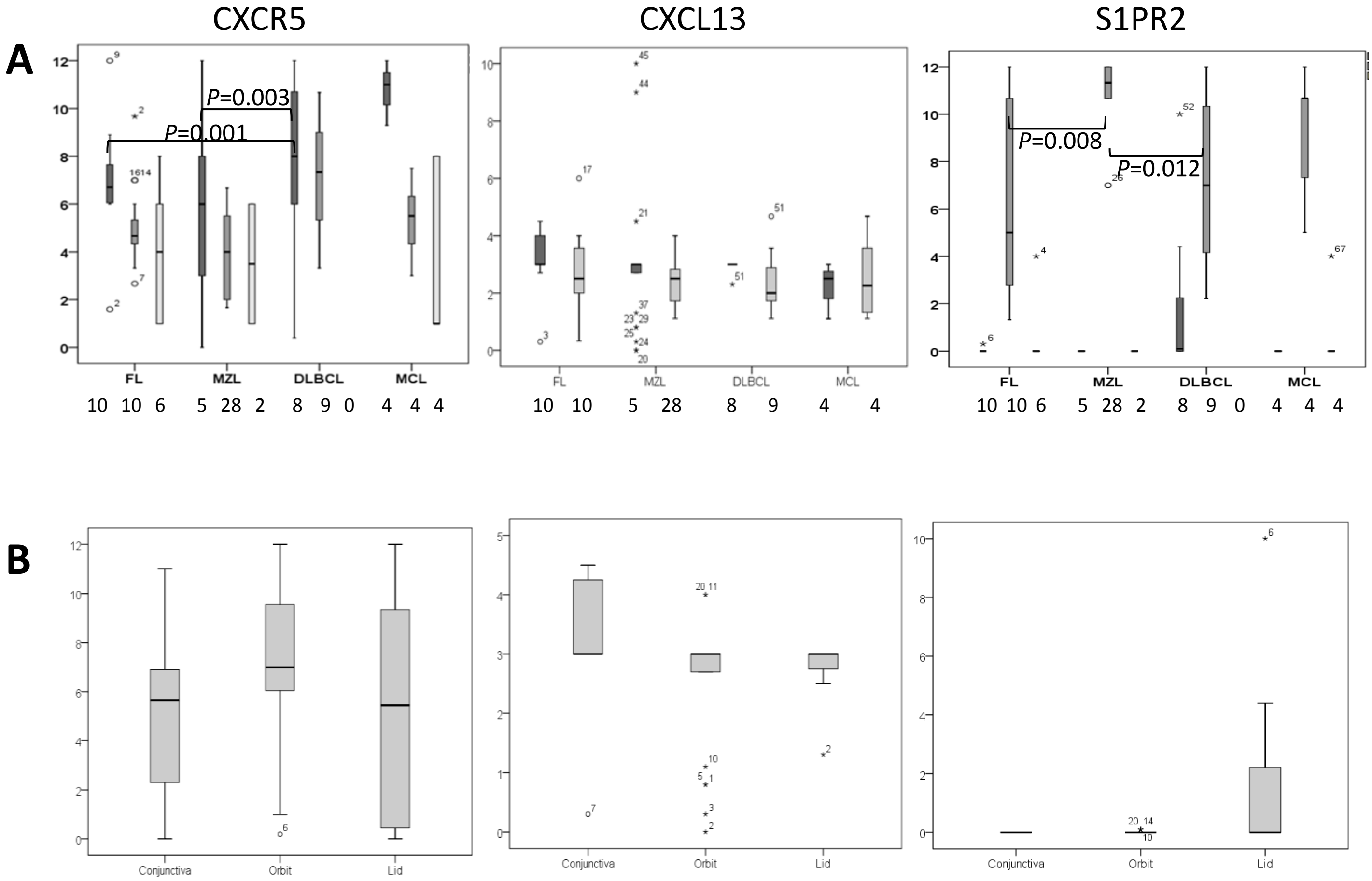

<u>Supplementary Figure 4. Expression of molecules involved in retention within LN.</u> A. Box and whiskers plots of the individual histological subtypes in the ocular adnexa ■, SLO ■, and leukaemic ■. Numbers below subtype indicate the number of patient samples. B. Box and whiskers plots of OLA at different locations within the ocular adnexa. Statistically significant differences in expression are shown.

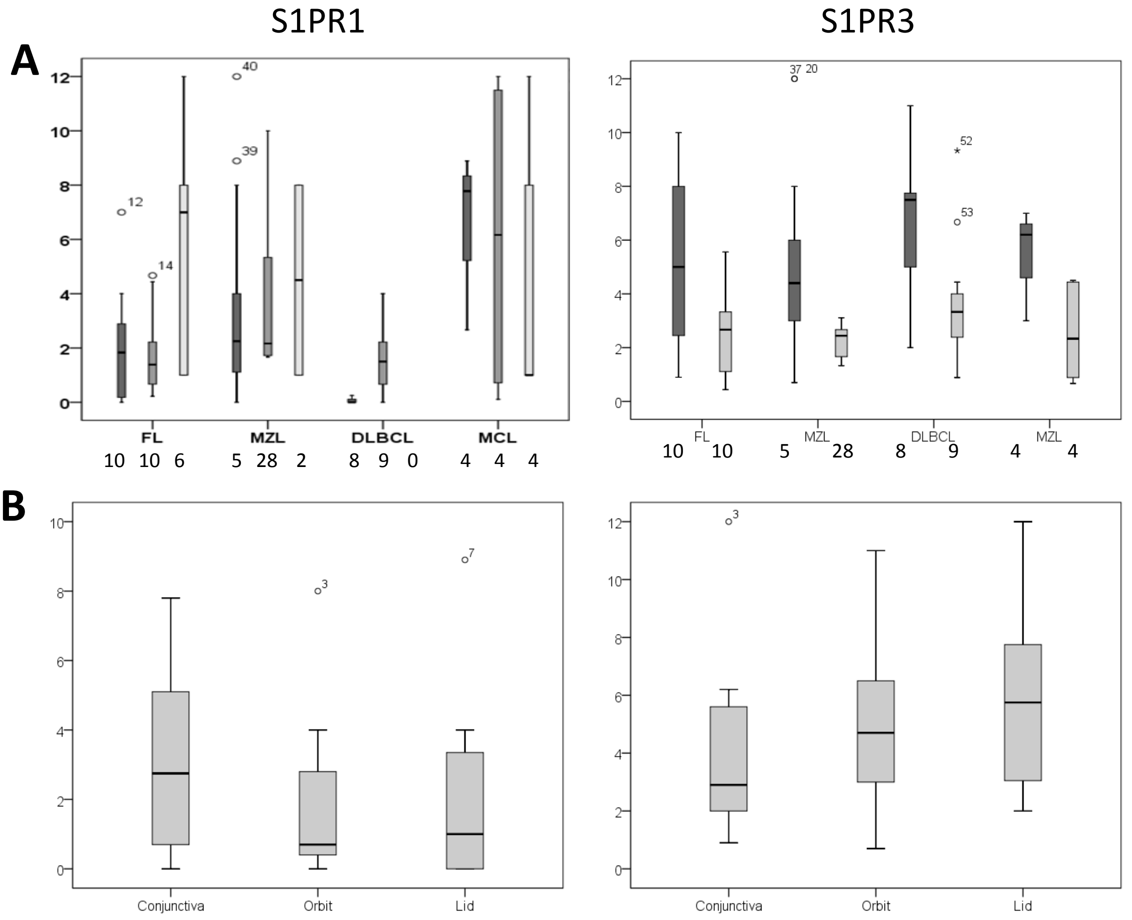

Supplementary Figure 5. Expression of molecules involved in egress from tissues. A. Box and whiskers plots of the individual histological subtypes in the ocular adnexa ■, SLO ■, and leukaemic ■. Numbers below subtype indicate the number of patient samples. B. Box and whiskers plots of OAL at different locations within the ocular adnexa. No statistically significant differences were seen.
